# Posterior Reversible Encephalopathy Syndrome Secondary to R-CHOP Chemotherapy Regimen

**DOI:** 10.7759/cureus.24988

**Published:** 2022-05-14

**Authors:** Selim Jennane, El Mehdi Mahtat, Mounir Ababou, Hicham El Maaroufi, Kamal Doghmi

**Affiliations:** 1 Clinical Hematology, Mohammed V Military Teaching Hospital, Mohammed V University, Rabat, MAR

**Keywords:** rituximab, r-chop chemotherapy, treatment dilemma, diffuse large b lymphoma, posterior reversible encephalopathy syndrome (pres), chemotherapy-related toxicity

## Abstract

Chemotherapy-induced posterior reversible encephalopathy (PRES) syndrome is a rare event. Its recurrence after reusing the incriminated molecules remains unpredictable. We report the case of a 58-year-old female patient being followed for a diffuse large B-cell lymphoma treated with rituximab, cyclophosphamide, hydroxydaunorubicin hydrochloride (doxorubicin hydrochloride), vincristine (Oncovin), and prednisone (R-CHOP) regimen. On the fourth day of the first R-CHOP cycle, the patient suddenly developed a headache, bilateral blurred vision, and drowsiness. The next day (day five), the patient had a spontaneously-resolving generalized tonic-clonic seizure associated with postictal bilateral blindness without any other neurological deficiency. Brain magnetic resonance imaging (MRI) revealed an increased bilateral signal intensity involving the cortex and subcortical white matter of the parietal and occipital lobes on the T2-weighted and the T2-weighted fluid-attenuated inversion recovery (FLAIR), which confirmed the diagnosis of PRES) syndrome. After resolution of symptoms, the continuation of the R-CHOP regimen did not lead to a recurrence of the syndrome.

## Introduction

Posterior reversible encephalopathy (PRES) syndrome is a clinico-radiological entity due to the sudden onset of reversible vasogenic edema of the white matter predominating in the posterior parieto-occipital regions [1]. This vasogenic edema is usually due to a sudden increase in blood pressure in a hypertensive patient. It can also occur in the absence of hypertension by direct endothelial toxicity of a cytotoxic agent [2]. We report a case of a 58-year-old woman who presented with PRES after receiving a widely used first-line chemotherapy regimen for B-cell non-Hodgkin lymphomas: the rituximab, cyclophosphamide, hydroxydaunorubicin hydrochloride (doxorubicin hydrochloride), vincristine (Oncovin), and prednisone (R-CHOP) regimen.

## Case presentation

A 58-year-old woman, with no history of known hypertension, was admitted to our department for the initial management of diffuse large B-cell lymphoma (DLBCL). liver hypermetabolism. According to the Ann Arbor classification, the patient was classified as stage IVB (hepatic involvement without central nervous system or bone marrow involvement) and R-CHOP regimen was proposed. At the time of her first cycle, the patient was in good general condition, conscious, afebrile, with a normal neurological examination. Blood pressure (BP) before the treatment was 110/70 mmHg. The first cycle was received over a period of two days. On the first day, the patient received pre-medication with methylprednisolone (80 mg) and paracetamol (1000 mg) followed by a six-hour infusion of rituximab. On the second day, she received the R-CHOP regimen. During these two days, the BP was monitored every four hours. The systolic blood pressure ranged between 95 and 130 mmHg and the diastolic blood pressure ranged between 61 mmHg and 85 mmHg. In addition, she concomitantly received anti-viral prophylaxis with valaciclovir (500 mg once a day), antithrombotic prophylaxis with enoxaparin (4000 IU per day), and intravenous antiemetic treatment with metoclopramide (10 mg four times a day) and ondansetron (8 mg twice a day).

**Figure 1:**
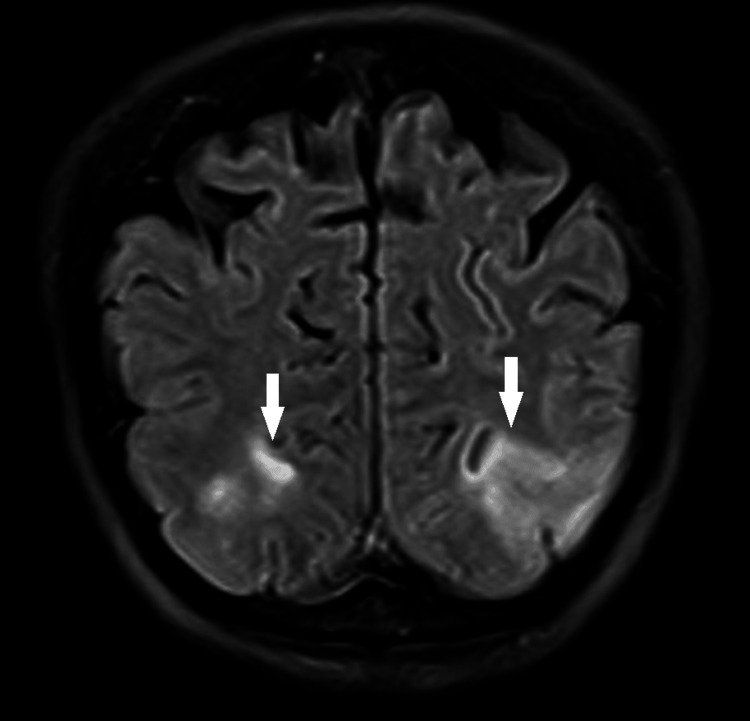
Coronal plane of brain magnetic resonance imaging (MRI) showing an increased bilateral signal intensity involving the cortex and subcortical white matter of the occipital lobe (Arrows) on the T2-weighted fluid-attenuated inversion recovery (FLAIR).

On the fourth day of the first R-CHOP cycle, the patient suddenly developed a headache, a bilateral blurred vision with right eye/oculus dextrus (OD) 3/10 and left eye/oculus sinister (OS) 5/10, and drowsiness. BP and fundus examination were normal. The next day (day five), the patient had a spontaneously resolving generalized tonic-clonic seizure associated with postictal bilateral blindness without any other neurological deficiency. BP was 140/90 mmHg. Brain computed tomography angiography (CTA) was normal. Brain magnetic resonance imaging (MRI) revealed an increased bilateral signal intensity involving the cortex and subcortical white matter of the parietal and occipital lobes on the T2-weighted and the T2-weighted fluid-attenuated inversion recovery (FLAIR), which confirmed the diagnosis of PRES (Figure 1).

The etiological workup of the PRES did not reveal electrolyte disorders (normal serum calcium, magnesium, and blood sugar levels) or biological signs of a post-chemotherapy tumor lysis syndrome (creatinine, serum potassium, phosphorus, and uric acid levels were normal), there was no evidence of liver or kidney impairment, no biological findings of thrombotic microangiopathy (no biological evidence of hemolysis and no schizocyte on the blood smear), and no clinical or biological signs of autoimmune disease or vasculitis (anti-nuclear antibodies and anti-neutrophil cytoplasmic antibodies were negative). Viral serological tests (human immunodeficiency virus and hepatitis B and C) were negative.

The progress was characterized by a slow and spontaneous improvement of the symptomatology without specific treatment while maintaining anti-viral and anti-thrombotic prophylaxis and antiemetic treatments in addition to an anti-epileptic drug. The visual disturbance resolved progressively in 11 days.

Considering the clinical decrease in the size and number of lymphadenopathies after one cycle, we decided to continue the same regimen. After six R-CHOP cycles, the end-of-treatment positron emission tomography (PET)-CT showed complete metabolic remission, and no evidence of PRES recurrence was observed throughout the treatment period.

## Discussion

Chemotherapy-induced PRES is a rare and unpredictable event [1]. Its pathophysiology is complex and various molecules have been incriminated. Experiments on mice have shown that an intracarotid infusion of cisplatin, a frequent cause of PRES, increased the permeability of the blood-brain barrier [3]. Bleomycin, a cytotoxic agent widely used in Hodgkin's lymphomas, causes a direct dose-dependent endothelial toxicity in vitro [4]. Some cytotoxic agents are able to activate an immune cascade involving the endothelial cell, thus increasing the permeability of the blood-brain barrier [5]. Chemotherapy could also improve the immune system's recognition of tumor cells and increase the production of cytokines that induce vascular instability at the blood-brain barrier [5].

Rituximab is an anti-CD20 monoclonal antibody that does not cross the blood-brain barrier. CD20 is expressed by all B lymphocytes but also by activated endothelial cells. Rituximab is therefore thought to cause direct endothelial toxicity and endothelin-induced vasospasm [6,7]. Post-chemotherapy PRES, therefore, involves a set of direct and indirect mechanisms that cause blood-brain barrier dysfunction [1]. Currently, only fifteen cases of post CHOP or R-CHOP PRES have been reported [2,8-10] even though the RCHOP regimen is the gold standard in the first-line treatment of DLBCL.

In contrast to other etiologies, hypertension is less common in chemotherapy-induced PRES [2]. Similarly, our patient had no history of high blood pressure. Reusing the same agents that are suspected to be the cause of PRES is a complex decision and there is little data in the literature dealing with this subject. In our case, the good clinical response after a single R-CHOP cycle encouraged us to pursue the same regimen. According to our literature review, three cases of post-CHOP or R-CHOP PRES describing subsequent therapeutic management have been reported. The authors of these three cases proposed changes in the chemotherapy regimen to prevent the recurrence of the syndrome [8-10]. In the first case, molecules frequently associated with PRES such as vincristine, cyclophosphamide, and granulocyte growth factors were withdrawn, but the patient experienced lymphoma progression after six cycles [8]. In the second case, the patient received a salvage regimen involving aracytin and etoposide followed by autologous stem-cell transplantation, which represents a more aggressive and therefore a more toxic treatment strategy [9]. In the third case, the authors proposed radiotherapy alone [10], which is not currently recommended in the treatment of disseminated DLBCL. Our observation shows that the continuation of the same regimen is risky, but does not systematically lead to the recurrence of PRES.

## Conclusions

This observation shows that despite its rarity, PRES syndrome can occur following a chemotherapy regimen as widely used as R-CHOP. Resumption of R-CHOP did not lead to the recurrence of PRES in our patient, but other larger studies should be conducted to identify criteria for resuming treatment after such a serious adverse event.
